# Treatment of Severe Asthma: Case Report of Fast Action of Mepolizumab in a Patient with Recent SARS-CoV-2 Infection

**DOI:** 10.3390/life14091063

**Published:** 2024-08-25

**Authors:** Cristiana Indolfi, Giulio Dinardo, Angela Klain, Fabio Decimo, Michele Miraglia del Giudice

**Affiliations:** Department of Woman, Child and of General and Specialized Surgery, University of Campania Luigi Vanvitelli, 80138 Naples, Italy; cristiana.indolfi@policliniconapoli.it (C.I.); fabio.decimo@unicampania.it (F.D.); michele.miragliadelgiudice@unicampania.it (M.M.d.G.)

**Keywords:** respiratory hypersensitivity, asthma, severe asthma, spirometry, FeNO, children, mepolizumab, SARS-CoV-2, case report

## Abstract

Asthma is one of the most common chronic inflammatory diseases of childhood with a heterogeneous impact on health and quality of life. Mepolizumab is an antagonist of interleukin-5, indicated as an adjunct therapy for severe refractory eosinophilic asthma in adolescents and children aged >6 years old. We present the case of a 9 year-old boy with severe asthma who experienced several asthmatic exacerbations following a SARS-CoV-2 infection, necessitating therapy with short-acting bronchodilators, oral corticosteroids, and hospitalization. We follow the patient using validated questionnaires for the evaluation of asthma control: Children Asthma Control Test, Asthma Control Questionnaire, respiratory function tests, and evaluation of exhaled nitric oxide fraction. After 12 weeks from the start of therapy with mepolizumab, we found significant improvements in lung function, a reduction in the degree of bronchial inflammation, and improvements in quality of life. No asthmatic exacerbations have been reported since the initiation of treatment with mepolizumab. Respiratory infections, such as those related to SARS-CoV-2, represent a significant risk factor for exacerbations in patients with moderate to severe forms of asthma. In our experience, following new episodes of exacerbation, the initiation of treatment with mepolizumab has allowed us to improve asthma control and enhance the quality of life of patients from the first doses. Although mepolizumab showed promise in this child with severe asthma during SARS-CoV-2 infection, the results from this single case cannot be generalized. Further studies are needed to confirm its safety and effectiveness.

## 1. Introduction

Asthma is a complex, chronic respiratory condition characterized by airway inflammation and hyperresponsiveness, leading to symptoms such as recurrent coughing, wheezing, chest tightness, and shortness of breath. It is the most prevalent chronic respiratory disorder among children, affecting approximately 8–10% of children worldwide [1,2]. Respiratory viral infections in early childhood have been identified as potential risk factors for developing asthma. Longitudinal studies have demonstrated a link between acute wheezing illnesses in infancy caused by viruses such as rhinovirus or respiratory syncytial virus (RSV) and the subsequent development of asthma in later childhood and adolescence [3,4,5]. A pivotal study published in 2002 highlighted the heightened risk of severe outcomes for individuals with asthma when infected with common cold viruses, including coronaviruses, which are the second most common cause of colds in humans [6]. Asthma exacerbations can be induced by a range of factors, with infections being a primary trigger. Asthma exacerbations are a leading cause of hospitalization in children and can be life-threatening if not properly managed, increasing the risk of developing chronic obstructive pulmonary disease (COPD) in adulthood [7,8,9]. The severity of virus-induced asthma exacerbations is closely related to how well the asthma is controlled [4].

### 1.1. COVID-19 and Pediatric Asthma

**Figure 1:**
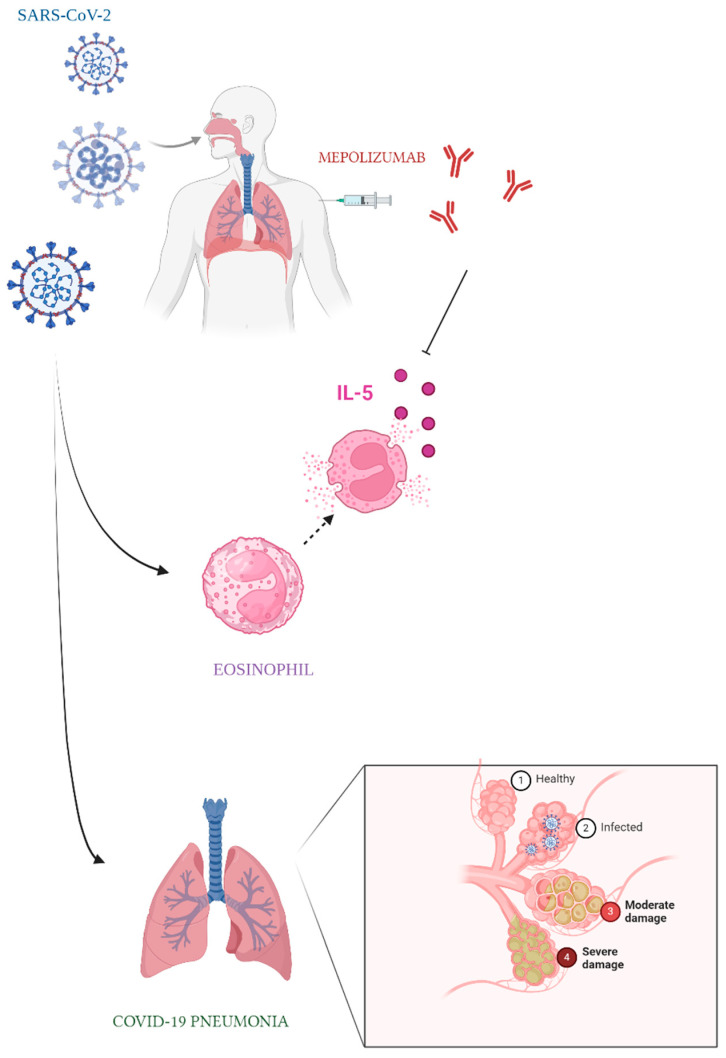
The interplay between asthma and SARS-CoV-2 infection in a patient undergoing biological therapy for asthma (mepolizumab).

In late 2019, a highly pathogenic strain of coronavirus, known as severe acute respiratory syndrome coronavirus 2 (SARS-CoV-2), was first identified in Wuhan, China. The virus rapidly spread across the globe, and by March 2020, the World Health Organization (WHO) officially declared COVID-19 disease a global pandemic. This virus can cause severe lower respiratory tract illnesses, such as pneumonia and acute respiratory distress syndrome [10,11]. The COVID-19 pandemic has significantly affected pediatric health, manifesting in immediate and long-term consequences, known as long COVID. Acute COVID-19 has been linked to various respiratory conditions in children, including the alteration of spirometry parameters [12,13]. Long COVID, a condition characterized by persistent symptoms following the initial infection, has emerged as a concerning issue among pediatric patients [14,15]. Parisi et al. conducted a cross-sectional survey that revealed a range of long-term sequelae, such as fatigue, respiratory problems, and neurological symptoms [16]. Beyond the physical health impacts, the pandemic has also exacerbated social and psychological issues among the pediatric population. The lockdowns and restrictions led to changes in children’s eating behaviours, noting an increase in unhealthy eating patterns during these periods and behavioural and sleep disorders, in particular in adolescents [17,18,19]. Asthma has been identified as a common underlying condition in some pediatric COVID-19 patients, but its impact on the severity of COVID-19 in children remains unclear [20,21]. Some studies suggest that asthma may increase the risk of severe COVID-19 outcomes in children. For example, Graff et al. found a significant association between asthma and the need for hospital admission and respiratory support in pediatric COVID-19 patients [22]. Another study by Kara et al. indicated that children with asthma were more likely to experience moderate to severe COVID-19 symptoms, highlighting the need for further investigation into asthma as a potential risk factor for severe COVID-19 in the pediatric population [23]. On the other side, subsequent studies have not consistently identified asthma as a significant comorbidity for COVID-19. Initial case series from China did not list asthma as a risk factor for severe COVID-19. Several large cohort studies found that asthmatic patients might actually have a reduced risk of severe COVID-19 outcomes [8]. A recent meta-analysis involving over 161,000 COVID-19 patients reported that asthma was present in only 1.6% of cases, a much lower prevalence than in the general population [24]. While the relationship between asthma and COVID-19 severity has been extensively explored, the impact of SARS-CoV-2 infection on the occurrence and exacerbation of asthma itself has not been clearly explained. The COVID-19 pandemic has had a mixed impact on the occurrence of asthma. On one hand, lockdowns and reduced exposure to outdoor air pollution have led to fewer asthma exacerbations in some individuals, as reported in a retrospective cohort study [25]. Conversely, the stress and anxiety associated with the pandemic, as well as potential viral infections, may have worsened asthma symptoms in others. Children who were hospitalized for asthma during the pandemic were generally more ill, requiring more intensive care and treatments [25]. Additionally, changes in healthcare access and treatment adherence during the pandemic could have influenced asthma management. In a recent study involving 104 patients, a notable correlation was identified between BMI and both COVID-19 symptoms and LUS scores (*p*-value < 0.05), whereas no correlations were detected with asthma or atopy [26]. The complex interplay between SARS-CoV-2 infection, asthma, and pediatric health requires further investigation to fully understand the implications of COVID-19 on asthma occurrence and management (Figure 1).

### 1.2. Management and Treatment of Severe Asthma

According to the latest GINA (Global Initiative for Asthma) document, asthma can be classified as mild, moderate, or severe [2]. The definition of severe asthma is defined as uncontrolled asthma despite adherence to a maximum optimized high-dose inhaled corticosteroids (ICSs) and long-acting beta2-agonists (LABA) treatment and management of contributing factors. It also includes asthma, which worsens when a high-dose treatment is reduced [9]. Childhood severe asthma may have a prevalence of up to 5% [27,28,29,30]. Asthma exacerbations can be triggered by several factors, including infections, insufficient use of asthma control medications, and exposure to allergens or pollutants. Typically, more than one factor contributes to these exacerbations, particularly in severe cases. Viral infections are notably critical, causing up to 90% of exacerbations, especially during the fall and spring in temperate climates when respiratory infections are prevalent. Additionally, these viral infections significantly influence seasonal rises in asthma attacks that align with the return of children to school after summer and spring breaks [4,31]. The GINA document states that the addiction to a biological drug should be taken into consideration in adolescents and children with severe uncontrolled asthma or who need to use oral corticosteroids (OCSs) frequently or continuously (which corresponds to step 5 in the guidelines) [2]. Mepolizumab is a humanized monoclonal antibody (Mab) with an IL-5 target, the main interleukin responsible for the maturation, activation, and increased survival of eosinophils [32,33,34]. Medullary recruitment and activation of eosinophils take place through the link between IL-5 and its receptor complex (IL-5R) at the surface level of eosinophil granulocytes. For this reason, IL-5R is a crucial therapeutic target in eosinophilic asthma [35]. Mepolizumab is indicated in adolescents and children aged 6 years or older as an additional therapy for severe asthma eosinophils with a history of at least 2 exacerbations of asthma despite maximum inhalation therapy in the previous 12 months or having received continuous oral steroid therapy in addition to maximum inhalation therapy, for at least 6 months in the last year. It is indicated when the following criteria are satisfied: eosinophilic count in peripheral blood 150 eosinophils/mmc in current determination and at least 300 eosinophils/mmc in previous year. In adults and adolescents aged 12 years, the recommended dose of mepolizumab is 100 mg subcutaneously once every 4 weeks. In children aged 6 to 11, the recommended dose of mepolizumab is 40 mg subcutaneously once every 4 weeks. Mepolizumab is available in a liquid formulation contained in a pen or pre-filled syringe and can be self-administered by the patient or administered by a care provider if the specialist determines that this is appropriate and whether the patient or the care provider is trained in injection techniques [35,36].

## 2. Materials and Methods

Our case report describes a child with severe allergic asthma who, following a SARS-CoV-2 infection, experienced a new episode of asthma exacerbation that led to an emergency room visit. Following hospital discharge, it was decided to initiate therapy with mepolizumab. At the baseline visit, a detailed medical history was recorded, and the patient underwent a physical examination, skin prick tests (SPT), and lung function tests. The SPT was performed following the standards set by the European Academy of Allergy and Clinical Immunology (EAACI) [37,38]. The allergen panel included house dust mites (*Dermatophagoides farinae* and *Dermatophagoides pteronyssinus*), cat, dog, grass mix, mugwort, ragweed, Parietaria officinalis, birch, hazel, olive tree, cypress, Alternaria tenuis, and Aspergillus mix (Lofarma, Milan, Italy). A histamine solution (10 mg/mL) served as the positive control, and a physiological solution was used as the negative control. The resulting wheals were measured by averaging the largest and smallest diameters, with a mean diameter of 5 mm or more indicating a positive result. Lung function was assessed using spirometry, interpreted according to the European Respiratory Society (ERS) guidelines [39]. The patient was asked to take a deep breath and then exhale forcefully and quickly, repeating this process three times with normal breaths in between. The level of asthma symptom control was assessed using the validated Children Asthma Control Test (C-ACT) and the Asthma Control Questionnaire (ACQ-7) [40]. The C-ACT evaluates clinical symptoms, activity limitations, use of short-acting β2-agonists, and airway constriction. The ACQ-7 includes seven items: five addressing symptoms, one concerning the use of rescue bronchodilators, and one measuring the forced expiratory volume in one second (FEV1% of the predicted normal) [40]. The patient is a 9 year-old boy with severe allergic asthma and allergic rhinitis sensitized exclusively to dust mites as assessed by the skin prick test (*Dermatophagoides pteronyssinus* 11 mm and *Dermatophagoides farinae* 8 mm). Sensitization to dust mites was also evaluated in the blood using the ImmunoCAP IgE assays (Thermo Fisher Scientific/Phadia, Uppsala, Sweden), showing the following results: Der p 1 41.7 kUA/L, Der p 2 54.4 kUA/L, Der p 23 16.9 kUA/L. A result >0.10 kUA/L is considered positive according to the technical data sheet. At the time of blood sampling, the patient had an eosinophil count of 600 cells/µL. The evaluation of exhaled nitric oxide before starting treatment with mepolizumab showed a value of 19 ppb. Before the asthma exacerbation, the patient was on high-dose steroid therapy, Fluticasone 500 µg/day, Salmeterol 100 µg/day, Montelukast 5 mg/day, and antihistamine therapy. Following the SARS-CoV-2 infection, the patient experienced a new asthma exacerbation that led to an emergency room visit. Subsequently, after hospital discharge, the patient started biological therapy with Mepolizumab 40 mg every 4 weeks as per the technical data sheet [41]. The patient had not undergone previous biological therapy.

## 3. Results

**Figure 2:**
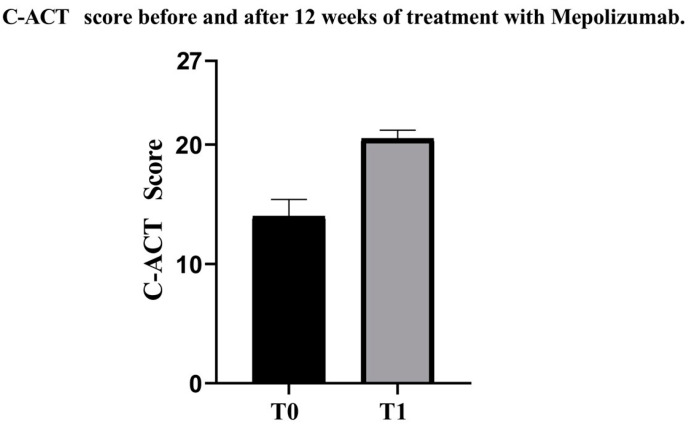
C-ACT score before and after 12 weeks of treatment with mepolizumab. C-ACT: Children Asthma Control Test; T0: Before treatment with mepolizumab; T1: After 12 weeks of treatment with mepolizumab.

**Figure 3:**
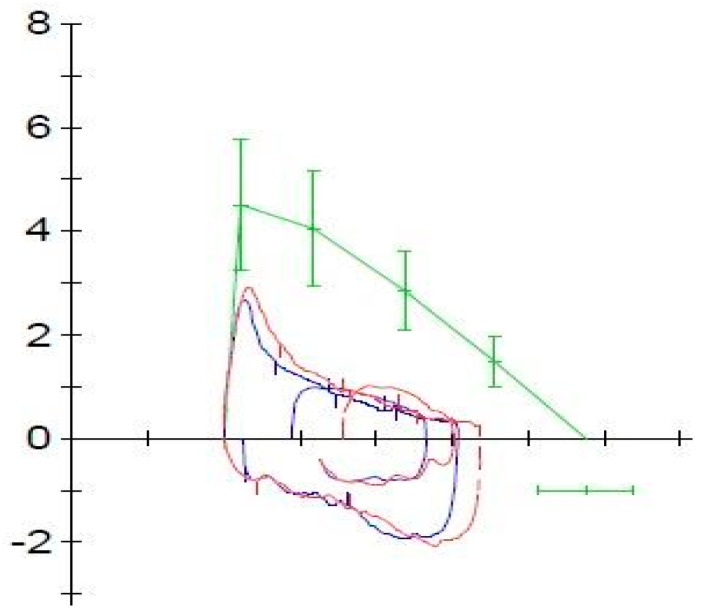
Spirometry before treatment with mepolizumab.

**Figure 4:**
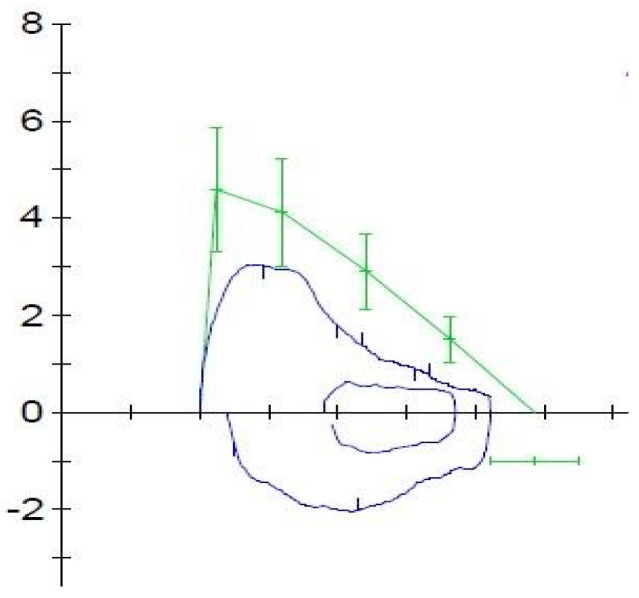
Spirometry after 12 weeks of treatment with mepolizumab.

During our experience, following the initiation of mepolizumab therapy, the patient showed significant improvements in both clinical-instrumental results and quality of life within 12 weeks. After initiation of therapy, the patient no longer had asthma exacerbations and no longer needed to take oral corticosteroids or administer short-acting bronchodilators. The patient’s C-ACT score showed significant improvement. At baseline, the C-ACT score was 15, indicating uncontrolled asthma. After 12 weeks of Mepolizumab treatment, the score increased to 21, reflecting a transition to partially controlled asthma (Figure 2). The ACQ-7 score, also demonstrated significant improvement (Table 1). Spirometry measurements demonstrated substantial enhancements in lung function. The FEV1 increased from 1.07 L (54% of the predicted value) to 1.69 L (83% of the predicted value) (Figure 3 and Figure 4). Similarly, the Forced Expiratory Flow at 25% of the pulmonary volume (FEF 25%) improved significantly from 1.47 L per second (36% of the predicted value) to 3.02 L per second (74% of the predicted value). Additionally, the Forced Expiratory Flow at 50% (FEF 50%) showed an increase from 0.85 L per second (30% of the predicted value) to 1.79 L per second (62% of the predicted value). Even the Forced Expiratory Flow at 75% (FEF 75%) increased from 0.60 L per second (41% of the predicted value) to 0.90 L per second (61% of the predicted value) (Table 1). These improvements indicate a significant enhancement in the patient’s pulmonary function following the introduction of Mepolizumab. The Fractional Exhaled Nitric Oxide (FeNO) levels, an indicator of eosinophilic airway inflammation, also showed a notable decrease. Initially, FeNO levels were measured at 19 parts per billion (ppb). After 12 weeks of treatment, these levels dropped to 3 ppb, suggesting a marked reduction in bronchial inflammation (Table 1). No adverse reactions have been reported after the starting of therapy with mepolizumab.

## 4. Discussion

Allergic severe asthma is a rare condition, affecting approximately 5% of individuals with asthma [2]. The literature contains only a limited number of clinical studies regarding the use of mepolizumab in pediatric patients [36]. This real-life case report presents the rapid and significant improvement of a pediatric patient with severe uncontrolled asthma following the initiation of mepolizumab therapy after a recent SARS-CoV-2 infection. The patient, a 9 year-old boy, exhibited marked improvements in both clinical and instrumental parameters within 12 weeks of treatment. Notably, there was a significant reduction in asthma exacerbations, leading to the discontinuation of oral corticosteroids and short-acting bronchodilators. The patient’s C-ACT score improved from 15 (uncontrolled asthma) to 21 (partially controlled asthma), and spirometry results showed an over 30% improvement in FEV1. Additionally, the FeNO levels decreased from 19 ppb to 3 ppb, indicating a reduction in bronchial inflammation. While most children with SARS-CoV-2 exhibit asymptomatic or mild symptoms, the interplay between COVID-19 and asthma, particularly severe cases, remains underexplored [12,42,43]. There is limited evidence on the prognostic implications of allergic diseases and asthma in the context of COVID-19, especially regarding long-term sequelae in pediatric patients [16,44]. Previous studies have focused primarily on adults, demonstrating a higher incidence of mild to moderate COVID-19 among those with severe asthma receiving biological treatments [45]. In the case series presented by Azim et al., four patients on mepolizumab treatment who contracted COVID-19 are discussed. Only one patient required hospital admission and oxygen therapy. Notably, the patients in their series differ from the case we described, as they were adults with multiple underlying health conditions. The article underscores the potential for mepolizumab to rapidly stabilize severe allergic asthma, even in the context of a recent SARS-CoV-2 infection [46]. While the observed improvements in this patient suggest that mepolizumab may hold potential as a therapeutic option for children with severe allergic asthma, particularly when exacerbated by respiratory infections like COVID-19, it is important to acknowledge the limitations of this case report. The positive outcome in this single case does not allow for generalization, and the absence of adverse reactions, though promising, cannot be definitively attributed to the treatment without further investigation. Therefore, larger, controlled clinical trials are necessary to validate these findings and to develop comprehensive guidelines for managing similar cases.

## 5. Conclusions

The disease linked to SARS-CoV-2 infection could trigger asthma exacerbations in patients with moderate-to-severe asthma and poor symptom control. Despite the experience of a single case, which will have to be confirmed through further studies, we observed that this drug proved to be safe, effective, and quick in improving asthma control. The patient from the beginning of therapy did not have new asthmatic exacerbations, and already after 12 weeks from the start of treatment, the patient had a significant increase in the parameters of respiratory function and improvement of the quality of life. Future research, including controlled clinical trials, is necessary to confirm the safety and efficacy of biological drugs in a broader population of asthmatic patients during SARS-CoV-2 infection. Another important area of research could be exploring the underlying mechanisms by which SARS-CoV-2 exacerbates asthma and how biological drugs, in particular mepolizumab, interact with those pathways to provide therapeutic benefits. Finally, comparative studies could evaluate mepolizumab against other biological therapies to determine its relative effectiveness and safety in asthmatic COVID-19 patients.

## Figures and Tables

**Table 1:** Comparison of values before and after 12 weeks of treatment with mepolizumab.

	Before Treatment with Mepolizumab	After 12 Weeks of Treatment with Mepolizumab
C-ACT	15	21
ACQ-7	2, 28	1, 14
FEV 1 L (%)	1.07 (54)	1.69 (83)
FEF 25% L/s (%)	1.47 (36)	3.02 (74)
FEF 50% L/s (%)	0.85 (30)	1.79 (62)
FEF 75% L/s (%)	0.60 (41)	0.90 (61)
FeNO	19	3

C-ACT: Children Asthma Control Test, ACQ: Asthma Control Questionnaire, FeNO: Exhaled nitric oxide fraction, FEV 1: forced expiratory volume in 1 s.

## Data Availability

The datasets used during the current study are available from the corresponding author on reasonable request.
